# Family language policy in a transnational family living in Finland: multilingual repertoire, language practices, and child agency

**DOI:** 10.3389/fpsyg.2024.1405411

**Published:** 2024-05-09

**Authors:** Olga Nenonen

**Affiliations:** Language Studies Unit, Faculty of Information Technology and Communication Sciences, Tampere University, Tampere, Finland

**Keywords:** family language policy, transnational family, language practices, child agency, multilingual repertoire, multilingualism

## Abstract

Each multilingual transnational family is unique and thus deserves to be carefully studied in terms of its family language policy (FLP). Speaker-centered approaches can provide a deeper understanding of linguistic diversity in a multilingual setting. The studied Russian-Italian family is raising a multilingual boy (8:2) in Finland. The multilingual repertoire includes Russian, Italian, Finnish, English, and Hebrew. In this case-study, an ethnographic approach is used to explore the multilingual family repertoire by presenting their lived experiences and language practices. I discuss the FLP and child’s active role in shaping the family’s linguistic practices (child agency). The following methods were combined: semi-structured interviews, language background surveys, written diary entries, self-recordings of interactions in the family, and a language portrait that depicts the child’s multilingual repertoire. The interviews and other recordings were transcribed manually. The following research questions guided the study: (1) How do the family members describe their FLP? (2) How does the FLP evolve through everyday interactions (language practices)? (3) How does the child exercise his agency in the family setting? The results reveal that the family’s language practices follow predominantly an one person-one language (OPOL) strategy; consequently, the child speaks a different language with each parent. However, the analysis of the language ideologies reveals positive attitudes toward both multilingualism and all the languages in the family’s repertoire, which explains the multilingual practices having multiplicity and unexpectedness. FLP is shaping the family language practices. Evidence of language hierarchy can be explained by a number of family-external and family-internal social factors.

## 1 Introduction

In the era of contemporary globalization, characterized by increased immigration and a rising number of intercultural marriages, the expanding variety of multilingual transnational families offers a significant challenge to researchers investigating family multilingualism. The unique nature of each family demands careful examination using the framework of family language policy (FLP) studies (Lanza, 2021). Speaker-centered approaches become vitally important to attain a profound comprehension of the linguistic diversity inherent in multilingual settings.

Parents in multilingual families make substantial efforts in raising their children bilingually, often striving for additive bilingualism. However, challenges arise, leading to reported frustrations when parents encounter difficulties transmitting their language to their children, resulting in children becoming passive bilinguals and rarely achieving balanced bilingualism (Protassova, 2018). Despite these challenges, a spectrum of experiences exists within multilingual families, some succeeding in nurturing bilingual children with high-level proficiency in both languages. It is crucial to grasp “the success stories” to understand how certain families manage to effectively raise bilingual children (Schwarz and Verschik, 2013). This article presents a qualitative case study, highlighting a “success story” within the present diverse landscape.

I depart from the idea that the family’s role in shaping the bilingualism of children is pivotal (Fishman, 1991; Lanza, 2007; Spolsky, 2012). That is why FLP studies are crucially important. Building on previous research, which originated from language policy studies, the domain of FLP merges the aspects of child language acquisition, language socialization, and language maintenance and shift (Curdt-Christiansen, 2018). Spolsky (2004) viewed language policy as a framework comprising three key aspects: language practices, language ideology, and language management. Language practices involve the regular selection of linguistic varieties of a repertoire reflecting the linguistic choices made by individuals or communities in everyday communication. Language ideology pertains to beliefs and attitudes regarding language and its use. Language management entails efforts to change or influence language practices through interventions or planning within a given context. Spolsky (2012) advocated for FLP being one of the critical domains of language policy. In recent years, extensive studies on FLP have been conducted, resulting in an abundance of literature, including books, special journal issues, and articles (for an overview of the field, see, e.g., Lanza and Gomes, 2020).

Earlier research on FLP primarily focused on language maintenance and shift, communication difficulties, and family experiences (Hua and Wei, 2016; Lanza and Gomes, 2020), with no special focus on the nuanced experiences within families. To address this gap, To address this gap, an ethnographic approach has been employed to explore the multilingual family repertoires, presenting experiences and language practices (Lanza, 2021). Thus, the shift in recent FLP research emphasizes issues related to lived experiences, agency (including child agency), and identity issues within multilingual families, while exploring bottom-up language policies emerging from everyday practices within the family (Hua and Wei, 2016; King, 2016; Lanza and Gomes, 2020; Smith-Christmas, 2020; Lanza, 2021). Recognizing bilingualism and multilingualism as experiences necessitates a holistic and multidimensional approach, contextualizing overall patterns within the broader coverage of the multilingual speakers, families, and communities involved (Hua and Wei, 2016, 665).

As I start this exploration of a specific multilingual transnational family, I aim to contribute to the evolving understanding of FLP, uncovering the dynamics of linguistic practices, agency, and identity construction within the familial context. Through a qualitative case study methodology, I unfold the layers of this family’s multilingual repertoire, providing insights into the complexity of the language dynamics in their daily lives.

The following research questions guide the study:

1. How do the family members describe their FLP?
2. How does FLP evolve through everyday interactions (language practices)?
3. How does the child exercise his agency in the family setting?

## 2 Methods

This ongoing case-study research project explores the dynamics of language practices, agency, and identity construction within a multilingual transnational family residing in Finland. The longitudinal study spans from 2019 to the present, unraveling the evolving language practices and dynamics over time (Lanza and Gomes, 2020).

The multilingual transnational family that I study represents families that “stretch across borders” (Baldassar et al., 2014, 169). Because of new types of mobility and communication technologies, their social relationships extend across time and place (Baldassar et al., 2014, 174). The focal family comprises first- and second-generation immigrants, embodying the essence of intercultural marriage. Having settled in Finland 11 years ago, the Russian-Italian family is raising a multilingual boy. Anonymity for participants is aimed for, the proper names were replaced with random letters (aliases), which enables the researcher to preserve the internal coherence of the data. The family includes a Russian-born mother (M) (44), an Italian-born father (P) (59), and a Finnish-born son (J) (8:2). The mother, a master of Arts and a teacher, is currently unemployed, while the father, with an incomplete bachelor’s degree, works in a restaurant.

An ethnographic perspective (Atkinson, 2007) allows us to explore FLP over time, and the analysis draws in the multilingual family repertoire by presenting their lived experiences and language practices. I also discuss the child’s active role in shaping the family’s linguistic practices—child agency. I combined the following methods to study the complexity of FLP: semi-structured interviews (collected in English), language background surveys, written diary entries (made in Russian) (Tseitlin et al., 2022, 198–220), self-recordings of interactions in the family, and a language portrait that provides bodily and emotional dimensions to the speaker’s multilingual repertoire (Kusters and De Meulder, 2019; Purkarthofer, 2019; Lanza, 2021). The interviews and other recordings were transcribed manually, and the content analysis was implemented to look for patterns of responses.

The metalanguaging data (speaker’s commentaries on his/her language practices as lived experience) from all tree family members were also documented: “Metalanguaging data are useful because the process of individuals trying to make sense of their world, in this case, language users reflecting on the linguistic performances by themselves as well as the others they are interacting with, is an integral part of the analytical process” (Hua and Wei, 2016, 658).

In adherence with ethical standards, informed consent has been acquired from all research participants.

## 3 Results

### 3.1 RQ1: how do the family members describe their family language policy?

#### 3.1.1 Multilingual repertoire and language practices

The family’s multilingual repertoire, as reported in semi-structured interviews, language background surveys, and written diary entries, encompasses the following languages:

- Russian [P: “(My) Russian was strong from the beginning, (I) wanted to interact with M’s relatives, still sometimes feel uncertain in Russian.”].
- Italian [M: “(My Italian is) not very strong probably…, Italian is good for shouting.”] (P: “J speaks Italian and Russian emotionally.”).
- English (P: “English is the *lingua franca*, emotionless, neutral language, and artificial language learnt from books.”).
- Finnish.
- Hebrew.

Notably, both parents learned each other’s languages at home, with the father furthering his proficiency in Russian through university-level courses. The son attends Jewish School of Helsinki, a comprehensive school where he has Finnish language classes 7 h a week, Hebrew 3 h a week, and English 1 h a week. He attends Italian lessons (the home language) at the comprehensive school for 2 h a week. He attended a private Russian school before, and now he attends a Russian complementary school for 3 h a week (Russian language, reading in Russian, and mathematics). He learned to read in Russian at the age 3:6 and started to read in Italian at the age 3:8. The son is thus engaged in multiple language classes, reflecting the family’s commitment to maintaining their linguistic diversity.

The family’s multilingual repertoire features Russian and Italian at the core, reflecting the parental linguistic backgrounds. English serves as a neutral *lingua franca*, with Finnish and Hebrew on the periphery. This configuration is integral to the family’s communication strategies, shaped by internal and external influencing factors. The family’s strategies for maintaining multilingualism encompass a great number of aspects, including formal and informal education, communication settings, the roles of parents, grandparents, and other people around, and ideologies. Noteworthy is the family’s proactive approach to transnational connections, fostering a positive environment for language maintenance.

The family’s transnational connections are evident through regular visits to St. Petersburg, representing the mother’s hometown (M: “St. Petersburg is my home; in Finland I feel myself a tourist”). The father is proud of his ability to speak Russian, emphasizing its importance for interacting with the mother’s relatives. Additionally, the family’s ties to Italy involved frequent visits before the COVID-19 pandemic, highlighting the impact of global events on their mobility.

#### 3.1.2 Family language planning

##### 3.1.2.1 Early strategies

Family language planning plays a vital role in the language policy of the focal family (this information was gathered from the semi-structured interviews and diary entries). Initially, the one person one language (OPOL) strategy was agreed upon and employed, involving a strict differentiation between the two first languages (L1s), Russian and Italian, for the first two and a half years of the child’s life. To ensure adequate exposure to both languages, the parents refrained from using English for interfamily communication. Efforts were concentrated on providing a rich input in both Russian and Italian. As the child reached 2.5 years, the introduction of additional languages, such as English, commenced through structured lessons facilitated by the mother.

##### 3.1.2.2 Language acquisition and societal integration

Recognizing the significance of societal integration, the family prioritized the child’s acquisition of Finnish, the language of the local society. Initial enrolment in a Finnish kindergarten (4:0) proved challenging, prompting a shift to a Jewish kindergarten and school (4:3) where Finnish is the primary medium of instruction. The family actively supports the child’s education in Finnish, emphasizing the importance of this societal language alongside ongoing efforts to develop proficiency in English and Hebrew.

##### 3.1.2.3 Flexible approaches to translanguaging

Over time, the family adopted a more flexible attitude toward translanguaging, allowing for language adjustments to attract attention or create humoristic effects. The father articulated a nuanced approach incorporating the OPOL strategy with adjustments, introducing Finnish when necessary, and occasionally employing Italian in the presence of others [P: “My guidelines are OPOL plus adjustment (+ Finnish), Italian only with J, if others are present—translation… sometimes we adjust, I think I found myself even speaking Finnish sometimes”]. The family maintains a positive spirit toward language learning, fostering a high level of multilingualism while remaining vigilant about the son’s L1 development [M: “If Finnish disturbs the language or other development (Finnish started at 3:8), then we will immediately leave the country”].

#### 3.1.3 Attitudes

##### 3.1.3.1 Satisfaction, pride, and positive feelings

The participants’ interviews offer evidence of attitudes toward multilingualism. The family exhibits high levels of satisfaction and pride in the son’s literacy levels in Russian and Italian aligning with age-appropriate benchmarks (M: “Everything went the ideal way, excellent, I’m proud of us!”). The mother, a language professional, imparts linguistic awareness to the child, fostering a creative and analytically adept approach to language. As a result, the son has acquired a profound linguistic awareness and practices a lot of linguistic analyses when trying to understand the meanings of words. He is also highly creative and invents new words based on one or several languages.

The mother expresses positive sentiments toward her hometown, St. Petersburg. She tells about visiting family, friends, and her alma mater (the Pedagogical University), and the prospect of returning to one’s “roots,” which reflects “a typical diasporic mentality of living in one place and thinking of (living in) another place, feeling a sense of belonging somewhere else” (Hua and Wei, 2016, 661–662). The father, despite weakened links to Italy, maintains a strong Italian identity. Both parents emphasize the cultural significance of language, viewing it as a practice intertwined with identity, happiness, and wisdom:

P: There are things that are more important than languages. As we have in an Italian song—“On the Doomsday English will be of no use.” No, I don’t make decisions based on languages, even with J, I mean I speak Italian because I want him to have that spirit in his soul to have that imprinting in his soul, which I connect the Italian language to sort of happiness, to a sort of wisdom somehow, a funny sort of wisdom or whatever and jokes and joyful living, I connect it to these things, and I would like J to have this imprinting, but then I’m not after the purity of the language actually.

##### 3.1.3.2 Pragmatic attitude to multilingualism

The parents pragmatically perceive multilingualism as conferring significant advantages on their son’s future. Beyond career prospects, they highlight enhanced confidence, additional benefits in various aspects of life, and the ability to view the world from diverse perspectives. This pragmatic stance underscores the broader societal advantages associated with multilingualism, aligning with the family’s commitment to fostering an open-minded and diverse worldview in the child.

In conclusion, the study results illuminate the intricate interplay of family language planning, language acquisition, and societal integration within a multilingual transnational family. The family’s strategy has changed over time from strict OPOL to a more flexible attitude, e.g., translanguaging. The flexible approaches, positive attitudes, and pragmatic recognition of the benefits of multilingualism contribute to a holistic understanding of language practices and their implications for individual and collective identities within the family unit.

### 3.2 RQ2: how does FLP evolve through everyday interactions (language practices)?

#### 3.2.1 Language practices in daily life

Based on interviews, language background surveys and diary entries, the research unveils the intricate language dynamics within the family’s everyday domestic interactions. Predominantly, Russian and Italian serve as the languages of communication at home, but the other languages in the family’s repertoire are integrated when feasible. Furthermore, at the present moment, the parents occasionally encourage the use of additional languages (Swedish and French) in daily conversations, enriching the multilingual environment within the household. This multilingual linguistic practice extends to hobbies, where the family cultivates multilingual engagement across various activities.

#### 3.2.2 Multilingual hobbies and activities

The son’s hobbies paint a vivid linguistic tapestry. Each language serves specific hobby domains, contributing to the child’s linguistic proficiency. Russian encompasses piano lessons, mathematics, calligraphy, chess, PC games, and reading with family and relatives. Italian finds its expression in chess, creating a unique bond between the father and the son. Finnish aligns with the violin lessons and school environment, while English manifests in the immersive realm of PC games, notably Minecraft as well as in English-language summer camps. Hebrew, primarily introduced as a school subject, is related to school events and celebrations, unveiling the multifaceted integration of languages into the child’s daily life. The family thus organizes the son’s hobbies to nurture his linguistic proficiency and consciously incorporate languages into the various leisure activities. J’s predisposition to explore new languages, e.g., Swedish and French, further enriches this linguistic repertoire.

#### 3.2.3 Transnational connections: St. Petersburg and beyond

The family is busy maintaining strong transnational ties and actively engages with friends and relatives in St. Petersburg, Italy, and Israel. These connections are not merely social but also extend to the mother’s alma mater, emphasizing the importance of academic and cultural links across borders.

In essence, the family’s language practices go beyond home communication, extending to the son’s hobbies, the family’s social connections, and transnational experiences. The exploration of the family’s language practices provides valuable insights into the diverse and dynamic ways multilingualism shapes the family’s daily life and the child’s language development.

### 3.3 RQ3: how does the child exercise his agency in the family setting?

The study delves into the influence of child agency within the FLP, employing the framework of Smith-Christmas (2020). This comprehensive framework incorporates various characteristics, such as linguistic norms, linguistic competence, compliance regimes, and generational positioning, offering a holistic approach to study the complex interplay of the child’s role in shaping family language practices.

#### 3.3.1 J’s impact on FLP: shaping habits

J is the focal point of the research; he actively shapes the FLP by exercising choices in the habitual modality. The implementation of the OPOL strategy in the family is notably influenced by J, who exhibits accuracy and persistence. His rejection of alternative linguistic practices, such as when M switched to English, exemplifies the child’s commitment to maintaining language boundaries within the family (J: “Mom speaks to dad some kind of nonsense”). On the other hand, being a strong adherent of OPOL does not prevent J from using languages other than Russian and Italian, thus J is highly creative; he plays with different languages, invents new words, writes poetry in English, and initiates multilingual games.

#### 3.3.2 J’s multifaceted language use and attitudes toward language learning

J’s language repertoire demonstrates a dynamic engagement with different languages based on contextual and interpersonal factors. Speaking Russian to M, Italian to P, Russian and Italian to relatives, and Russian, Finnish, and English to friends, J showcases a sophisticated navigation of linguistic choices influenced by relationships and environments. J’s attitudes toward language learning at school exhibits a spectrum of emotions. While he expresses contentment attending the Jewish School of Helsinki, he appears less enthused about the Russian complementary school due to the perceived workload challenges. This nuanced response reflects the child’s agency in negotiating his language learning experiences.

##### 3.3.2.1 Language portrait

The analysis of multilingual language users’ language portraits helps to investigate their backgrounds, lived experiences, environment, thoughts, attitudes, and feelings (Wei, 2011). I explore the language portrait made by J as well as the follow-up interview as a way of interpreting the portrait (see more in Busch, 2006).

J’s portrayal aligns with previous studies, using national flags to symbolize languages (Kress and Van Leeuwen, 2006). In his language portrait, J has depicted flags of Italy, the Russian Federation, and Finland, as well as the flags of the United States and the United Kingdom.

J: When I think about a language, I imagine a flag. At first, I thought about Italian, then about Russian, Finnish, and English. I used these flags for English because they speak English in these countries. I like these languages equally. And the national anthems too.

While J asserts the equal importance of all languages, the positioning of flags suggests a nuanced hierarchy, aligning with the core-to-periphery pattern observed in language portraits. Studies show that the languages in language portraits are depicted following a core-to-periphery pattern (e.g., Kusters and De Meulder, 2019; Kasap, 2021), thus the most significant languages, e.g., mother tongues, are colored in the head and the central parts of body like the heart or chest (Busch, 2006; Kasap, 2021). In J’s portrait, Italian and Russian, depicted in the head and main body parts, reveal their significant roles as mother tongues. Finnish and English, represented lower in the legs, convey their functional significance. J’s artistic choices provide meaningful insights into his perceived hierarchy of languages within his multilingual repertoire.

#### 3.3.3 Linguistic competence and awareness

J emerges as a linguistically adept individual, displaying not only a high command of grammatical structures and lexical items but also a profound linguistic awareness. Actively engaging in linguistic analyses and correcting family members’ pronunciation, J strives to uphold linguistic norms within the family context. J has acquired Finnish faster than his parents and it gives him the opportunity to act not only as a language specialist within the family but also as an interpreter. This type of language brokering (McQuillan and Tse, 1995; Antonini, 2016) occurs both at home and in public situations, which helps to socialize the parents in a better way into the sociocultural environment of the dominant language scenery.

In essence, the research unveils the intricate interplay of child agency in shaping the FLP, emphasizing the dynamic nature of linguistic choices, competencies, and attitudes within the familial and broader socio-cultural contexts. J’s journey is tangible proof of the multifaceted dimensions of language acquisition in the ever-evolving scenery of multilingualism.

## 4 Discussion

The purpose of this case-study is to explore the success story in a transnational family where the child becomes multilingual, multicultural, and multiliterate. I focus on a child who was exposed to Russian and Italian languages from birth and later acquired Finnish, English, and Hebrew. The research helps to unravel the dynamics in the examined FLP: the family’s language practices, initially characterized by a strict OPOL strategy, have gradually developed into a more flexible approach, incorporating translanguaging in certain contexts (as reported by family members themselves). Notably, the child prefers to use OPOL and adeptly switches home languages depending on the situation, mainly maintaining distinct linguistic interactions with each parent. The smooth transition from a rigid adherence to OPOL to a nuanced approach took place in the FLP, which aligns with the family’s engagement in translanguaging, highlighting the dynamic nature of their language practices.

The deviations from OPOL are particularly evident in the realms of school, hobbies, and communication outside the family. The departure from a strictly OPOL-based approach reflects the family’s adaptability to their multilingual environment. The family’s language ideologies play a key role in shaping these practices; they express positive attitudes toward multilingualism and each language of the family repertoire. This positive orientation contributes to the multilingual practices with a large number of languages, adding an element of unpredictability to everyday interactions.

The family’s language hierarchy is influenced by both external and internal social factors and reflects the importance of maintaining an ethnic and cultural identity. For this family, language, comprising both L1s, is not just a means of communication but also a cultural practice integral to preserving and developing their distinctive cultural identity. This emphasis on cultural identity takes precedence over other values in their language decisions, shaping their commitment to maintaining both heritage languages: Russian and Italian.

Despite the deviations from strict OPOL, the family’s efforts to preserve the heritage languages remain evident. The FLP and language practices correspond to each other, which underlines the link between intentional language planning and its attainment within the family context. This alignment highlights the relational and dynamic nature of child agency, as it is shaped by FLP and, reciprocally, influences language practices within the family. The study provides further evidence to the crucial role of child agency in FLP (Smith-Christmas, 2020; Zhan, 2023).

The family is living in a highly multilingual environment, and its positive language ideologies contribute to the high level of multilingualism observed. The emphasis on ethnic identity over other values in language decisions highlights the significance of cultural continuity within a diasporic context. Though living in Finland, the parents travel between memory and imagination (Hua and Wei, 2016), with the son demonstrating contentment in the present while maintaining a connection to his cultural roots. This intricate interplay of language practices, ideologies, and family dynamics elucidates the multifaceted nature of multilingualism within a transnational family context.

The results of the study are in line with previous research (e.g., Schwarz and Verschik, 2013): FLP outcomes are not solely influenced by the language policy, e.g., a strict attitude to the OPOL principle; various factors like individual language attitudes, feelings, ethnic identities, parents’ perceptions of language stability, opportunities for creative language use, and children’s views on multilingualism play significant roles.

This case study is limited by its small-scale nature, and the longitudinal work needs to be continued. Further research will extend not just to a larger period of time, but also to more detailed analyses of the large data sample, since research has revealed interesting findings about linguistic creativity (e.g., Rakhilina et al., 2016; Ringblom and Dobrova, 2019; Fridman and Meir, 2023) and metalanguaging data (Hua and Wei, 2016) provided by the members of the family. Systematic empirical investigation of the possible enrichment of the multilingual repertoire and changes in language hierarchy will be continued.

## Data availability statement

The original contributions presented in the study are included in the article/Supplementary material; further inquiries can be directed to the corresponding author.

## Ethics statement

Ethical approval was not required for the study involving human samples in accordance with the Ethical principles of research with human participants and ethical review in the human sciences in Finland issued by the Finnish National Board on Research Integrity (TENK) in 2019 because the study did not involve intervening in the physical integrity of research participants, did not expose research participants to exceptionally strong stimuli, and does not entail a security risk to the participants or their family members. Written informed consent for participation in this study was provided by the participants’ legal guardians/next of kin. Written informed consent was obtained from the individual(s), and minor(s)’ legal guardian/next of kin, for the publication of any potentially identifiable images or data included in this article.

## Author contributions

ON: Conceptualization, Data curation, Formal Analysis, Investigation, Methodology, Project administration, Writing – original draft.

## Supplementary material

SUPPLEMENTARY FIGURE 1 Language portrait made by J.
