# Supplementary figures and images for: Family language policy in a transnational family living in Finland: multilingual repertoire, language practices, and child agency
Source: Front Psychol. 2024 May 9;15:1405411. doi: 10.3389/fpsyg.2024.1405411 (PMC11112104; doi:10.3389/fpsyg.2024.1405411)

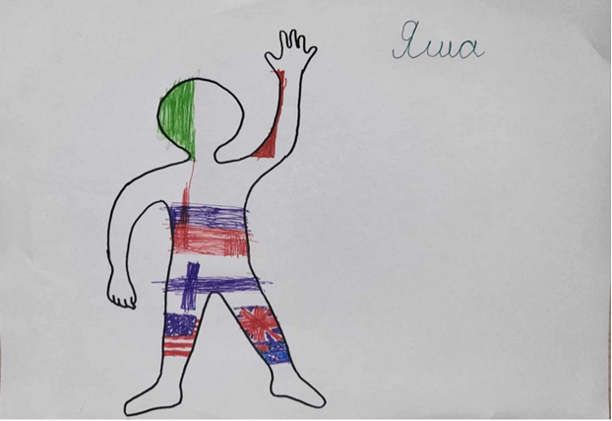

Supplement: SUPPLEMENTARY FIGURE 1 — Language portrait made by J. [file Image_1.TIF]
